# Residency Patterns and Migration Dynamics of Adult Bull Sharks (*Carcharhinus leucas*) on the East Coast of Southern Africa

**DOI:** 10.1371/journal.pone.0109357

**Published:** 2014-10-08

**Authors:** Ryan Daly, Malcolm J. Smale, Paul D. Cowley, Pierre W. Froneman

**Affiliations:** 1 Department of Zoology & Entomology, Rhodes University, Grahamstown, South Africa; 2 Port Elizabeth Museum at Bayworld, Humewood, Port Elizabeth, South Africa; 3 South African Institute for Aquatic Biodiversity (SAIAB), Grahamstown, South Africa; 4 Department of Zoology, Nelson Mandela Metropolitan University, Port Elizabeth, South Africa; Hawaii Pacific University, United States of America

## Abstract

Bull sharks (*Carcharhinus leucas*) are globally distributed top predators that play an important ecological role within coastal marine communities. However, little is known about the spatial and temporal scales of their habitat use and associated ecological role. In this study, we employed passive acoustic telemetry to investigate the residency patterns and migration dynamics of 18 adult bull sharks (195–283 cm total length) tagged in southern Mozambique for a period of between 10 and 22 months. The majority of sharks (n = 16) exhibited temporally and spatially variable residency patterns interspersed with migration events. Ten individuals undertook coastal migrations that ranged between 433 and 709 km (mean  = 533 km) with eight of these sharks returning to the study site. During migration, individuals exhibited rates of movement between 2 and 59 km.d^−1^ (mean  = 17.58 km.d^−1^) and were recorded travelling annual distances of between 450 and 3760 km (mean  = 1163 km). Migration towards lower latitudes primarily took place in austral spring and winter and there was a significant negative correlation between residency and mean monthly sea temperature at the study site. This suggested that seasonal change is the primary driver behind migration events but further investigation is required to assess how foraging and reproductive activity may influence residency patterns and migration. Results from this study highlight the need for further understanding of bull shark migration dynamics and suggest that effective conservation strategies for this vulnerable species necessitate the incorporation of congruent trans-boundary policies over large spatial scales.

## Introduction

The spatial ecology of apex predatory shark species is important to understand when considering their role in structuring marine communities and proposing effective conservation strategies [1], [2], [3], [4]. Large predatory shark species may exert broad scale influences on multiple levels of community structure through predation and associated risk affects [5], [6], [7]. Quantifying their movement patterns is important for understanding the associated spatial and temporal scales over which these sharks influence their marine communities [8]. Elucidating the role of top predatory sharks in marine communities is important as top marine predatory populations are increasingly under threat from over-exploitation [6], [9], [10].

Bull sharks (*Carcharhinus leucas*) are large (>3 m), globally distributed sharks that typically occur in tropical and warm temperate coastal marine environments [11]. Adult bull sharks are known to consume a variety of prey from multiple trophic levels [12], [13] and may influence marine communities through predation over a broad geographical range [13]. However, little is known about the long-term (>1 yr) temporal and spatial scales over which adult bull sharks typically move. Previous studies suggest that adult bull sharks exhibit high levels of residency interspersed with some degree of short to medium scale geographical migrations linked with seasonal change [11], [14], [15]. On the east coast of South Africa, bull sharks are known to undertake seasonal migrations into more temperate latitudes during summer [11], [16] but have been thought to be largely resident with limited home ranges [12], [17].

For a slow growing and late maturing species such as the bull shark [18], exhibiting periods of residency and undertaking large-scale migrations are important life history characteristics that may make the species especially vulnerable to both fishing pressure and habitat loss [19], [20]. Evidence suggests that bather protection nets have already depleted bull shark populations on the east coast of South Africa [12], [21] and understanding the residency patterns and migration dynamics of this species will help to mitigate future threats and improve conservation strategies [22], [23]. In this study, we employed passive acoustic telemetry to investigate the habitat use of adult bull sharks at a remote natural aggregation site in southern Mozambique that is minimally influenced by human activity. Our aim was to investigate the temporal and spatial dynamics of adult bull shark residency patterns at the study site and quantify the timing, frequency and distance of migration events.

## Methods

### Ethics statement

All research in this investigation was conducted under the permit number 0002/2010 issued by The Mozambican Directorate of National Conservation Areas. The Animal Ethics Committee of the Department of Zoology and Entomology at Rhodes University approved the research protocol used in this study (ethical clearance number ZOOL-14-2012).

### Study site

**Figure 1:**
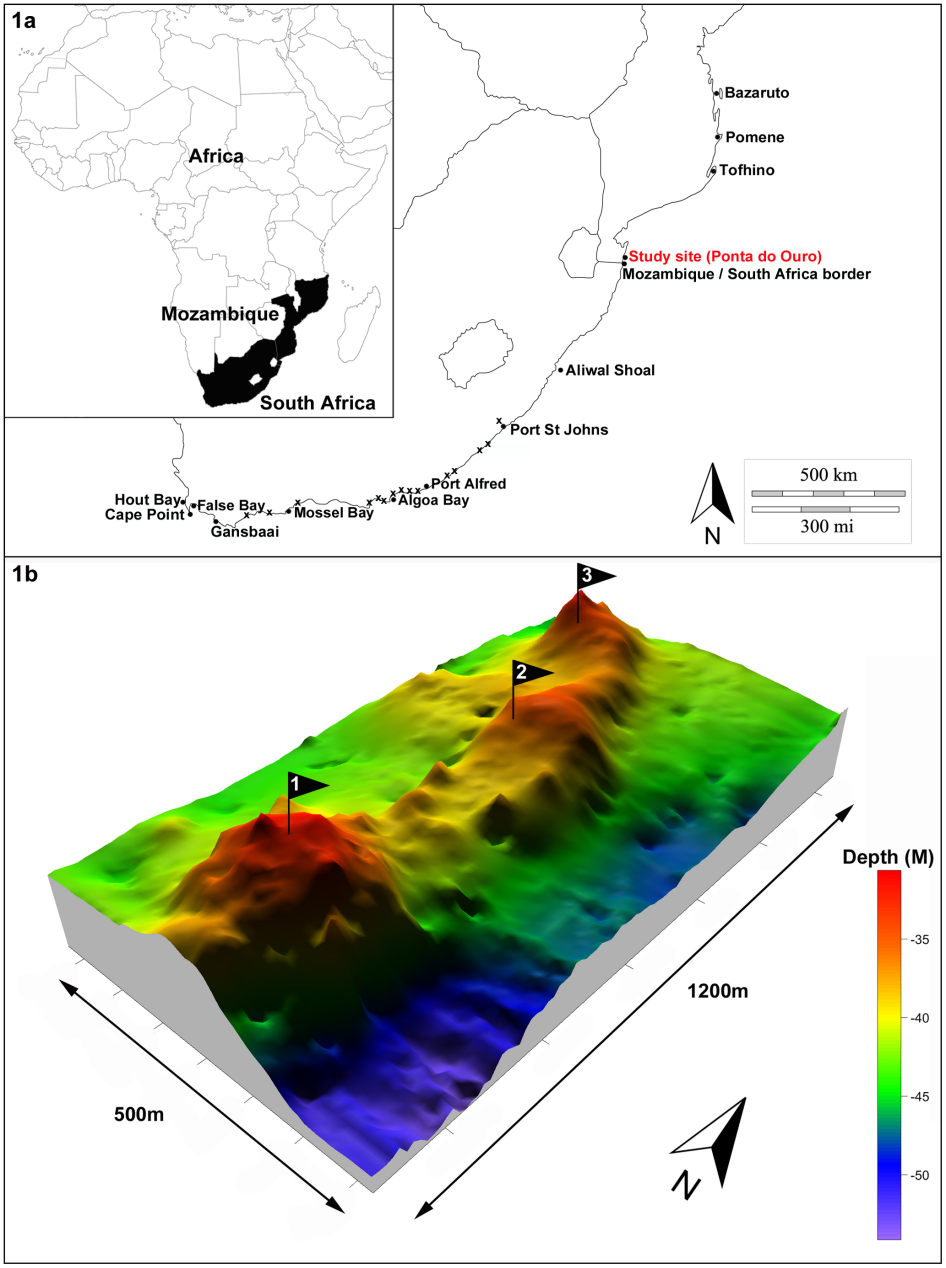
Study site Map and Pinnacle reef bathymetry. Figure 1a shows a map of southern Africa with the location of the study site at Ponta do Ouro in southern Mozambique. All other named locations represent acoustic receiver sites on the coast (represented by a circle) or within an estuary (represented by an x) from which additional bull shark detection data was obtained. Figure 1b shows the bathymetry of the Pinnacle reef complex with the colour scale indicating the corresponding depth of the reef showing the shallow spine surrounded by reef drop offs. Numbered flags represent the approximate location of three acoustic receivers on the reef.

This investigation took place in southern Mozambique within the Ponta do Ouro Partial Marine Reserve (Fig. 1a). This region of coastline lies in a biogeographical transition zone between the subtropical east coast of South Africa and the tropical coast of central Mozambique [24] referred to as the Delagoa bioregion [25]. The focal study site was a reef complex called the Pinnacle reef, located approximately 3.7 km offshore (S26° 44.900′ E32° 56.000′). The reef is a fossilized coastal dune [26] running parallel to the coast and consisting of a ridge approximately 1.2 km long with a series of shallower pinnacles along its spine (Fig. 1b). The reef sandstone substrate is interspersed with sand patches and has scattered alcyonarian coral growth with limited scleractinian coral growth [27].

### Shark capture and tagging

Between January 2012 and February 2013, 18 bull sharks were captured and tagged at the Pinnacle reef. Sharks were captured from a 6 m vessel using baited Mustad 18/o circle hooks connected to 450 pound carbon coated steel trace attached to 20 m of nylon rope and two 15-liter surface buoys. Once a shark was hooked, it was left to tire for 20–30 minutes before being brought alongside the vessel where it was inverted to induce a state of tonic immobility and then secured alongside the vessel while remaining partially submerged. Captured sharks were first measured (TL) and then surgically equipped with a VEMCO V16 coded acoustic transmitter (VEMCO Ltd., Halifax, Canada). The tags were implanted into the peritoneal cavity via a 2–3 cm incision in the abdominal wall that was subsequently sutured closed using a surgical needle and thread [28]. Tags were deployed in four main batches and all transmitted at 69 kHz at low power with a nominal delay of 120 seconds (range: 80–160 seconds). Before release, a uniquely coded plastic dart tag (ORI, South Africa) was externally attached to the base of the first dorsal fin to identify internally tagged individuals. To release the shark, the hook was removed from the mouth before turning the shark into an upright position and releasing it. The time from securing the shark alongside the vessel to its release ranged between 10 to 15 minutes. Tagged sharks ranged between 195 and 283 cm TL (mean TL  = 254.4 cm) with a M:F ratio of 11∶7 (Table 1). Based on the measured length, all sharks were considered sexually mature adults [12], [18] and all males (with the exception of ID # 13) had calcified claspers.

**Table 1:** Summary of all bull sharks tagged with acoustic transmitters at the Pinnacle reef.

Shark ID	Date tagged	TL (cm)	Sex	Total detections	Total number of days detected	Residency Index (RI)
1	21.01.2012	250	M	4135	172	0.26
2	23.01.2012	220	F	4649	86	0.13
3	30.01.2012	260	M	19	5	0.01
4	02.02.2012	280	F	117	15	0.02
5	23.11.2012	282	M	884	76	0.21
6	24.11.2012	270	M	4	1	<0.01
7	07.12.2012	253	F	24	7	<0.01
8	09.12.2012	244	M	1142	36	0.10
9	18.12.2012	263	M	1134	96	0.28
10	20.12.2012	264	M	1667	45	0.13
11	24.01.2013	250	F	1790	40	0.12
12	26.01.2013	262	M	128	8	0.03
13	29.01.2013	195	M	126	12	0.04
14	30.01.2013	251	F	145	21	0.04
15	31.01.2013	283	M	1677	76	0.26
16	03.02.2013	261	F	1546	44	0.15
17	18.02.2013	241	F	996	23	0.08
18	19.02.2013	251	M	10960	169	0.61

TL represents total length. Total detections refer to all detections on all receivers at the study site over the length of the study.

### Receiver deployment

An array of three VEMCO VR2 acoustic receivers was deployed at the study site (the Pinnacle reef) between November 2011 and January 2012. All receivers were attached directly to the reef substrate using a 4 mm stainless steel anchor chain connected to a 3 m long 14 mm nylon rope suspended by a 200 mm trawl float with the receiver attached vertically to the rope 2.5 m from the substrate. The deployment of three receivers on the Pinnacle reef occurred in locations where reef profile was low and within safe diving limits (Fig. 1b). These three receivers collected data continuously from November 2011 to November 2013. Two additional receivers were deployed inshore of the Pinnacle reef approximately 700 m from the shore and spaced 2.4 km apart. The inshore receivers were maintained from January 2012 to November 2013 although data were retrieved from only one inshore receiver due to equipment failure.

In addition to the local receiver array, data were also obtained from VEMCO VR2W acoustic receiver arrays along the coast and within estuaries in South Africa maintained by the Acoustic Tracking Array Platform (Hout Bay, False Bay, Gansbaai, Mossel Bay, Algoa Bay, Port Alfred, Dwesa MPA, Port St Johns, South Africa/Mozambique border) and the Marine Megafauna Foundation (Tofinho, Pomene and Bazaruto) as well as an array at Aliwal Shoal maintained by the Department of Environmental Affairs: Oceans and Coasts (Fig. 1a). All receivers at the above mentioned locations were operational over the same period as data were collected for this study.

Two temperature loggers (Hobo Water Temp Pro v2), set to record temperature every hour, were deployed at the Pinnacle reef from November 2011 until November 2013 (ongoing). One logger was attached to receiver number 1 (Fig. 1b) at 30 m depth approximately 2 m from the bottom. The second logger was located 3 km inshore of the Pinnacle reef at a depth of 16 m, approximately 2 m from the bottom.

### Range testing

Receiver range testing was conducted at the Pinnacle reef to help interpret the detection data at the study site [29], [30]. Results from the range test suggested that detection range at the study site was relatively poor (<100 m) but within the same range as previous studies using the same transmitters (VEMCO V16-6L) in similar environments [31], [32]. A full description of the range test experiment methods and results are presented in the supporting information of the manuscript (Text S1, Table S1, Figure S1). Based on the results from the range test it can be assumed that receivers 1–3 do not have overlapping detection ranges.

### Data analysis

Detection data from the Pinnacle reef study site was downloaded and initially analyzed using the software package VUE version 2.0.6 (VEMCO 2013). Data from all receivers were combined to investigate overall presence and absence at the study site. Daily detections were plotted on a time line. A shark was considered present on a certain day when at least one detection was recorded within 24 hours. Potentially false detection data were examined by considering realistic detection time frames and potential tag collisions and any erroneous data were removed. Combined daily detection data from all Pinnacle receivers for males and females were analyzed for differences using a Students t-test.

**Figure 2:**
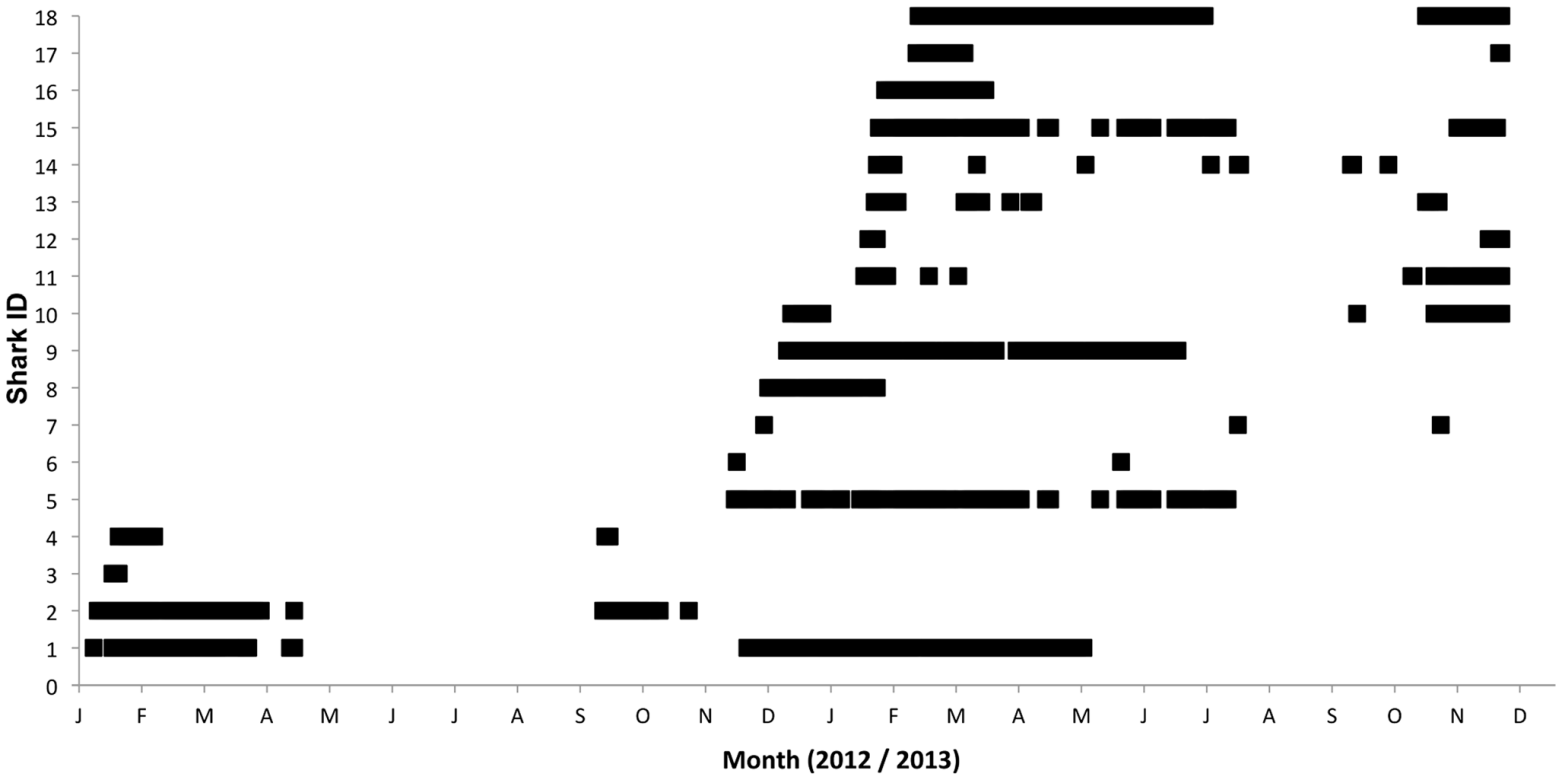
Daily bull shark detection data from all receivers at the study site. Detection data is represented by black squares and presented on a timeline from January 2012 to December 2014 for all 18 tagged sharks. The first detection data point for an individual shark corresponds to the day the shark was tagged at the study site.

Data from all three receivers at the Pinnacle reef were combined to investigate the temporal habitat use of individual bull sharks during periods of continuous presence (Fig 2). Detection data from individual sharks were binned into hourly periods and the total number of detections recorded within each hour of a 24-hour period was represented in rose diagrams using the statistical software package Oriana (version 4, Kovach Computing Services). Rao's Spacing Test [33] was employed to investigate if the temporal presence (represented by the number of detections recorded for each hour of a 24-hour period) of individual bull sharks at the Pinnacle reef was homogenous. To test for uniformity in the temporal detection data, Rao's Spacing Test investigates the null hypothesis that the recorded temporal detection data are evenly distributed throughout each hour of a 24-hour period. The level of statistical significance is determined from a table of simulated critical points [34] with the statistical significance set to p<0.05. The time of sunrise ranged between 04:51 h and 06:39 h and the time of sunset ranged between 17:07 h and 18:44 h during the length of this investigation.

Overall residency indices (RI) at the Pinnacle reef were calculated for each shark by dividing the number of days each shark was detected by the total number of days each shark was monitored [35], [36]. This measure standardizes the detection data for each shark regardless of the monitoring period by providing a figure of between 0 (shark detected on zero days that it was monitored) and 1 (shark detected on every day that it was monitored) that allows us to compare residency indices between sharks that were monitored over different time periods. An overall residency index was calculated for the entire study period, as well as the mean monthly indices. To investigate any potential bias between the monitoring periods of individual sharks and the calculated residency indices a regression analysis and one-way ANOVA were employed. The overall residency indices of males and females were compared using a Student's t-test. A single linkage hierarchical cluster analysis was also employed to investigate groupings within the sample population based on overall residency indices. Finally, a regression analysis and one-way ANOVA were employed to investigate the relationship between monthly residency indices and mean monthly sea temperatures.

Migration events were defined as directed persistent one-way movements between two receivers over large spatial scales [37]. The overall migration distances for sharks were measured using Garmin Homeport (version 2.2.1) and calculated as the minimum distance along the coast between receiver locations [38]. The rate of travel (km.day^−1^ and km.h^−1^) by a shark was calculated from the minimum distance moved between two receivers (km) divided by the time at liberty (days) during a migration event. Regression analyses with a one-way ANOVA were employed to investigate any relationships between shark length and migration distance and speed.

Unless otherwise stated, all data analyses were conducted using the statistical package R (CRAN 2009).

## Results

### Presence/absence at the study site

Bull sharks were monitored at the study site for 664 days between January 2012 and November 2013 (Fig. 2). All sharks were detected on the receiver array at the Pinnacle reef for at least one day after tagging. Individual tag detection totals ranged between 3 and 10958 detections (1724.1±2664.6, mean ± SD) over a period of 1 to 172 days (50.3±52.9, mean ± SD) (Table 1). Shark presence was variable with some sharks (ID # 1,2,5,8,9,15,16,18) exhibiting continuous periods of presence for over one month that ranged between 46 and 158 days (Fig. 2). Although there was not a significant difference (t-test, DF = 6, p>0.05) in the total number of days detected between males and females, males (62.8±62.0, mean ± SD), on average were detected for more days than females (mean = 30.6±27.9, mean ± SD). Most sharks (72%) were detected at the inshore receiver for an average of 5.8 (SD = 9.0) days but nearly all of these individuals (97.1%) were also detected at the Pinnacle reef on the same day.

The majority (15 out of 18) of tagged sharks were also detected at the array of five receivers located at the border of South Africa and Mozambique, approximately 12 km south of the Pinnacle reef. Detections at the border receiver array only occurred over periods when sharks were also detected at the Pinnacle reef without exceptions. The total number of days that sharks were detected on the border receiver array ranged between 1 and 102 days (16.8±29.3, mean ± SD) and the proportion of days that sharks were detected at both the border receiver array and the Pinnacle reef array over the same monitoring period ranged widely between 0.01% and 100% (49±41.6, mean ± SD).

### Temporal residency patterns

**Figure 3:**
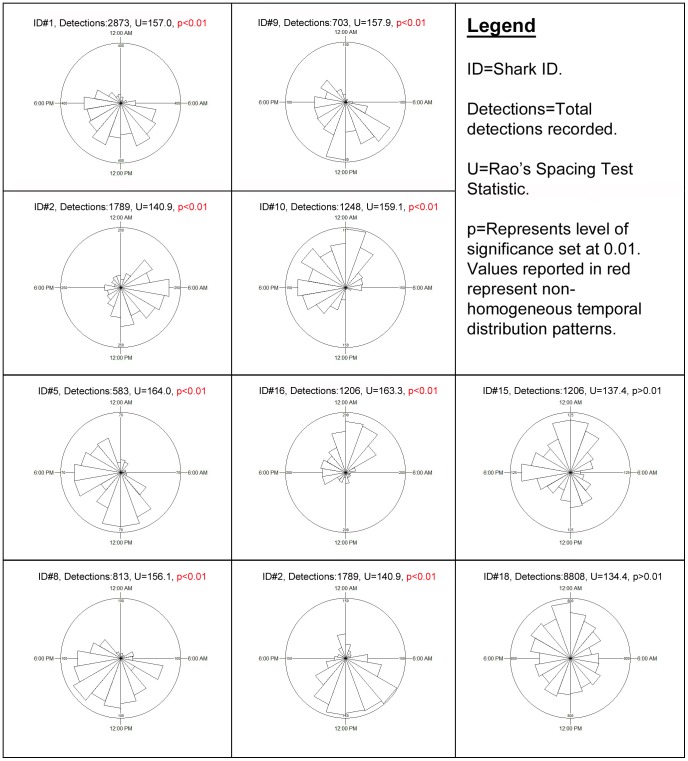
Circular plots showing the temporal detection distribution of bull sharks at the Pinnacle reef. Each plot represents the detection frequency within each hour of a 24-hour period. The temporal distribution of most sharks (except 15 and 18) was significantly non-homogenous (Rao's spacing test, p<0.01). Individual sharks not represented had insufficient data for the analysis.

Figure 3 represents the temporal detection patterns at the Pinnacle reef of all sharks with sufficient data (n = 10). Hourly detection frequencies for all sharks (except ID # 15 and 18) exhibited non-homogeneous distributions (Rao's spacing test, p<0.01). There was some overlap in temporal use of the Pinnacle reef between individuals (ID # 1, 2,5,8,9,16) that appeared to be present primarily from morning (∼7am) until early evening (∼8pm). Other individuals, however, were primarily present during the evening (ID # 10 and 11) whilst others were present throughout the day and night (ID # 15 and 18). Most sharks (except ID # 15 and 18) exhibited increased habitat use at the Pinnacle reef over a 12-hour period followed by a 12-hour period of absence.

### Residency

**Figure 4:**
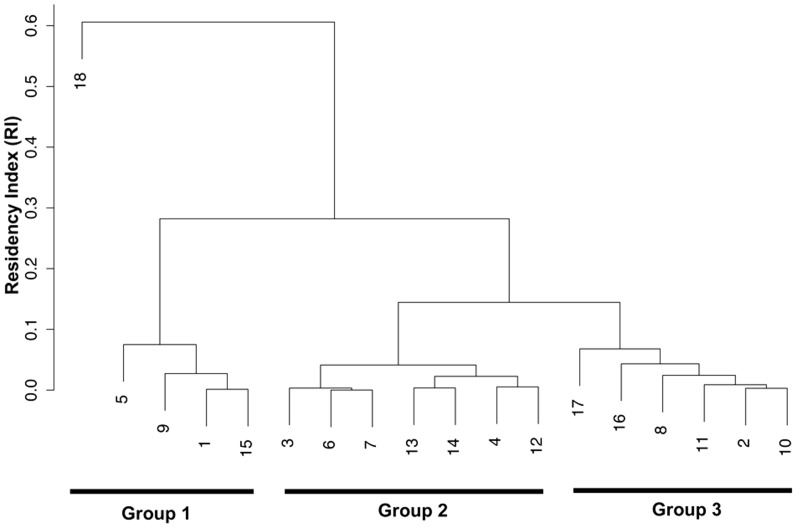
Cluster analysis results. Dendrogram showing the groupings of individual sharks based on their calculated residency indices at the study site.

Bull sharks monitored at the Pinnacle reef exhibited residency indices (RI) ranging between 0.001 and 0.67 (0.14±0.15, mean ± SD) over the duration of the study (Table 1). Results from a hierarchical cluster analysis (Fig. 4) suggest that there were three main groupings of sharks based on their overall residency indices. Five of 18 tagged sharks (ID # 18,5,1,9,15) exhibited relatively high overall residency indices (RI>0.2) with periods of prolonged (>3 consecutive months) high monthly mean residency indices (>0.3) interspersed with relatively short periods of absence (Fig. 2) and appeared to be seasonal residents. One individual (ID # 18) appeared as an outlier within the seasonal resident group with an overall residency index (RI = 0.6) substantially higher than other individuals within the group. The second group of seven individuals (ID # 3,6,7,13,14,4,12) exhibited very low resident indices (RI<0.1) with only a few detections over the entire study period (Fig. 2). The third group of sharks (ID # 17,16,8,11,2,10) exhibited intermediate residency indices (RI = 0.08–0.2) with periods (1–3 months) of high monthly mean residency indices (RI>0.3) interspersed with long periods of absence (Fig. 2).

**Figure 5:**
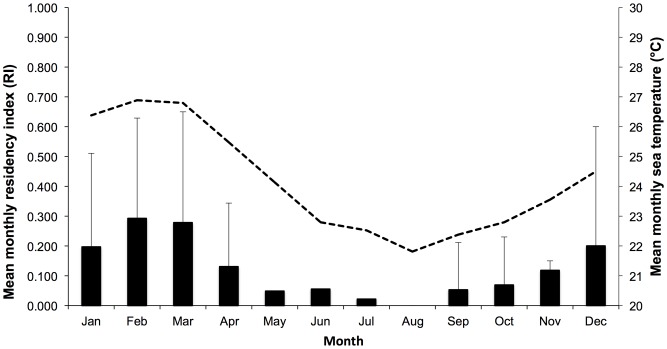
Mean monthly residency indices and mean monthly sea temperature. Bar charts represent the mean monthly residency indices from all tagged bull sharks detected at the Pinnacle reef. Error bars representing standard deviation. The dashed line represents the mean monthly sea temperatures (°C) recorded at the study site.

There were no significant relationships between the total monitoring periods of individual sharks and the total number of detections or total days detected or calculated residency indices (ANOVA, DF = 3, p>0.05). There was no significant (t-test, DF = 6, p>0.05) difference in the overall residency indices exhibited by female or male sharks, however, the mean male residency index (0.18±0.18, mean ± SD) was higher than the mean female residency index (0.08±0.06, mean ± SD) and only male sharks exhibited residency indices over 0.2 (n = 5). Monthly mean residency indices were highest during summer (Dec-Feb) (mean = 0.33) and autumn (Mar-May) (mean = 0.46), compared with winter (Jun-Aug) (mean = 0.08) and spring (Sep-Nov) (mean = 0.24), and showed a positive correlation (R^2^ = 0.84, ANOVA, DF = 1, p<0.05) with increased water temperature at the study site (Fig. 5).

### Migration

**Figure 6:**
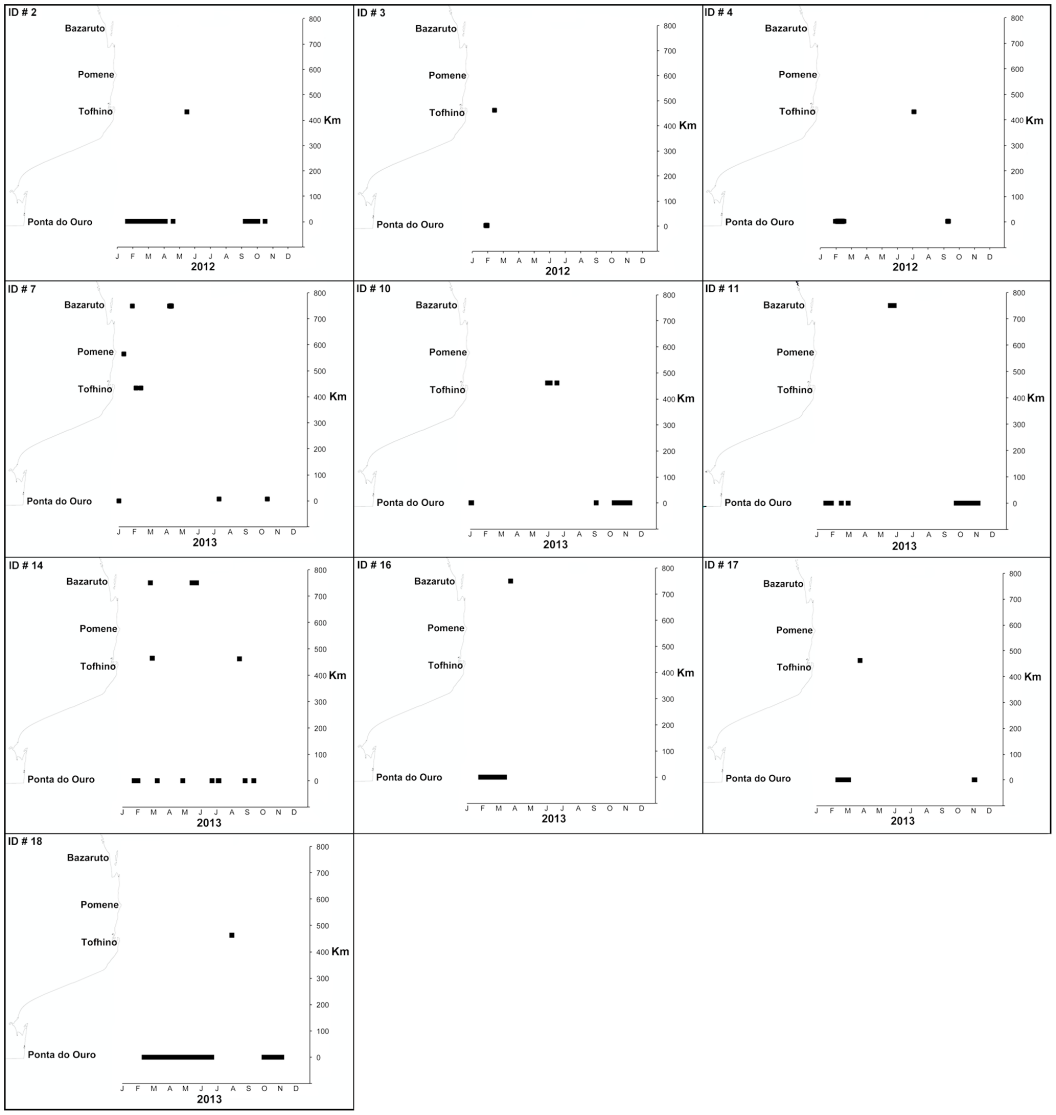
Bull shark migration events along the coast of Mozambique. The x-axis represents a time line showing months and year. The y-axis shows distance along the coast in kilometers (starting at Ponta do Ouro) corresponding with the coastal map of southern Mozambique. The solid square markings show annual bull shark detection events at the study site and other locations within Mozambique.

Ten out of 18 tagged sharks undertook large-scale migrations (>400 km) primarily in northerly direction (Fig. 6) with only 1 shark (ID # 7) undertaking a southward migration of 450 km to Aliwal Shoal, South Africa (Fig. 1a). No other detections were recorded at any of the receivers located in South African coastal waters (Fig. 1a). Eight out of ten sharks returned to the study site after migration events (ID # 2,4,7,10,11,14,17,18) and one shark undertook multiple return migrations (ID # 14). Individuals that undertook migrations ranged in size between 220 and 280 cm TL (mean  = 258.8 cm TL) and 70% were females. The minimum distance that sharks travelled during migration events (persistent and directed one-way movements between receivers) ranged between 433 km and 709 km (533±122.8, mean ± SD) with daily rates of movement ranging between 2.0 km.day^−1^ and 59.1 km.day^−1^ (17.6±15.5, mean ± SD) and with corresponding speed ranging between 0.08 and 2.5 km.h^−1^ (0.75±0.65, mean ± SD). Total annual minimum distances travelled by sharks ranged between 450 and 3760 km (mean  = 1163 km). There was no significant relationship (ANOVA, DF = 2, p>0.05) between total length and distance travelled or speed. Nor were there any significant relationships (ANOVA, DF = 2, p>0.05) between gender and distance travelled or speed, although females did exhibit a mean travelling distance (991.7 km±327.3 km, mean ± SD) which was greater than that of males (770 km±266.7 km, mean ± SD). Northward migration events took place between January and September with the majority (75%) during austral autumn and winter (March-August).

## Discussion

Evidence provided in this study suggests that the Pinnacle reef in southern Mozambique is an important aggregation site for adult bull sharks and that they exhibit prolonged periods of residency, primarily during the austral summer months. Importantly, this study highlights the geographical scale of movement that adult bull sharks are capable of undertaking along the coast of southern Africa, and suggests that they are not as sedentary as previously thought [12], [17]. This study also documents the first return migration events for the species along this coast and emphasizes the need for greater understanding of bull shark migration dynamics.

### Presence/absence

Data from this study suggests that the Pinnacle reef is an important habitat for adult bull sharks with tagged individuals exhibiting periods of prolonged presence (Fig. 2). Detection data from the nearby receiver array at the south African/Mozambican border also suggests that bull sharks at the study site do undertake medium-scale movements (<12 km) but the frequency, direction and range of these movements may vary widely between individuals. The majority of tagged sharks (72%) also undertook inshore forays at the study site but further investigation is required to determine the importance of inshore habitat use.

### Temporal residency patterns

The majority of tagged sharks appeared to exhibited diel patterns in habitat use during this investigation, which is consistent with previous observations of multiple shark species across a range of habitats [39], [40], [41]. Previous studies investigating diel patterns in bull shark habitat use found that sharks exhibited increased site fidelity during daylight hours followed by absence or broader habitat use at night [31]. Findings from this investigation showed that the majority of individuals appeared to demonstrate similar diel patterns, although some individuals exhibited higher site fidelity at night while others exhibited equal habitat use throughout a 24-hour cycle. The behavioral drivers behind diel habitat use may vary and include foraging [8], predator avoidance [42] and thermal regulation [43]. In the absence predator constraints, it is likely that foraging behavior is an important driver of temporal habitat use. The significantly biased and varied temporal habitat use patterns of sharks at the study site suggest that individuals may employ a variety of temporally segregated foraging strategies.

### Residency

The overall range of residency indices recorded for bull sharks during this study were lower than a previous investigation in Fiji [31], although in this study there was no food provisioning, the receiver array was smaller and the study site was located in an environment with greater seasonal change. There appeared to be individual variation in the levels of residency behavior that could be categorized into three general groupings (Fig. 4) but overall austral summer and autumn residency indices were substantially higher for individuals and mean monthly sea temperatures appeared to be a major driver of habitat use at the study site. Bull shark seasonal habitat use is well documented and may be more pronounced in sub-tropical environments such as the study site [12], [16], [44].

Although the seasonal change in water temperature appeared to be an important driver of residency at the study site, the wide range of residency indices suggests other drivers, such as reproductive activity and foraging strategies, may also be important factors determining habitat use by bull sharks [20], [45], [46], [47]. The onset of increasing levels of residency exhibited by bull sharks at the study site appeared to be associated with large aggregations of Carangid fish species (mainly *Caranx ignobilis* but also *Carangoides fulvoguttatus, Carangoides gymnostethus* and *Caranx sexfasciatus*), known to be bull shark prey items (Video S1) [12]. However, it was not clear if bull sharks were responding to increased prey abundance or if both the sharks and the fish aggregations were corresponding to environmental cues. Prey availability is a known driver of shark habitat use [48], [49], thus it is likely that the increase in seasonal potential prey abundance at the study site influences the foraging behavior and associated residency patterns of bull sharks at the study site.

### Migration

Evidence from this study confirmed that bull sharks are capable of substantial migrations [15] and provided the first description of the distance and speed bull sharks travel on return migration events. Migration distances recorded for individuals in this investigation were within range reported for bull sharks [15] but present the longest travel distances recorded for bull sharks in southern Africa. Previous studies in South Africa using conventional tagging [17] and catch data [12] showed that bull sharks were a sedentary species occupying small home ranges. In contrast, this investigation shows that it is more likely that bull sharks along the southern African coast exhibit periods of residency interspersed with substantial geographical migration events.

Most migration events recorded in this investigation were linked with seasonal change at the study site with a northward migration to warmer latitudes during austral winter and spring and a southward movement into more temperate latitudes during summer. This is consistent with previous literature that has shown bull sharks may be more abundant at higher latitudes along the east coast of South Africa during warmer austral summer months [12]. However, one shark (a mature female, ID # 14) undertook multiple return migration events over multiple seasons, suggesting that seasonal change may not be the only driver of migration events. Other large shark species may undertake migrations driven by reproductive activity [38] and prey availability [49]. As female sharks in this investigation were recorded migrating further and undertaking the majority of migration events (70%), it is possible that gender specific migration linked to reproductive cues were a factor that determined the distance, direction and frequency of migration events.

Evidence from this study also indicate that bull sharks are capable of covering substantial distances over short periods of time with sustained mean (0.74 km.h^−1^) and maximum (2.46 km.h^−1^) rates of movement faster than those previously recorded for bull sharks [15]. Recorded rates of movement were still, however, substantially slower than tiger (3–4 km.h^−1^) and white sharks (4.7 km.h^−1^) undertaking sustained movements [50], [51], [52] but showed that within a year, adult bull sharks are capable of covering a broad geographical range that may include coastal waters of multiple countries. This supports evidence from a previous investigation that showed adult bull sharks have a geographically broad foraging range along the east coast of southern Africa [13]. Although in this investigation bull sharks were only detected as far north as Bazaruto, it is possible that some sharks travel substantially further north and additional investigation is required to establish the extent of bull sharks northward range.

### Summary

Previous investigations using telemetry to monitor adult bull shark habitat use have shown that bull sharks have an affinity for coastal environments, exhibit periods of residency and are capable of substantial geographical migrations [14], [15], [31], [53]. Results of the current study generally supported these findings and provided further understanding of the residency patterns and migration dynamics of bull sharks in southern Africa with the first description of return migration events for the species. Our results suggest that coastal migrations are a frequent and important component of bull sharks habitat use and are primarily driven by seasonal change. Seasonal change is often the driver of animal migration but further investigation is required to understand how spatiotemporally fluctuating resources and reproductive activity influence bull shark residency patterns and migration dynamics [37]. Improving our understanding of bull shark migration dynamics is important to elucidate the spatial scales of their ecological role [54] and plan effective conservation management strategies for this species [23]. A broad range of management approaches encompassing trans-boundary cooperation may be required to effectively conserve critical bull shark habitat.

## Supporting Information

Figure S1 **Range test results.** Proportion of transmissions received vs the expected detections received represented as a percentage (0–100%) from tags deployed at the study site at varying distances, depths and times of day. Error bars represent standard deviation.
(TIF)

Table S1 **Range test results represented as mean detection rates expressed as percentages.** Distance corresponds to the distance of the receiver from the transmitter (5 m, 100 m, 200 m, 300 m, 400 m), Day or Night corresponds to the time of day detections were recorded and Surface (10 m) or Bottom (25 m) corresponds with the depth at which the transmitter was positioned in the water column. Standard deviations are presented in parenthesis.
(DOCX)

Text S1 **Range test experiment.** Methods, results and discussion of the range test experiment.
(DOCX)

Video S1 **Carangid fish aggregation video.** Video showing adult bull sharks associated with a Carangid fish (*Caranx ignobilis*) aggregation at the study site.
(MOV)

## References

[1] Speed CW, Field IC, Meekan MG, Bradshaw CJA (2010) Complexities of coastal shark movements and their implications for management. Marine Ecology Progress Series 408: 275–293.

[2] Abrantes KG, Barnett A (2011) Intrapopulation variations in diet and habitat use in a marine apex predator, the broadnose sevengill shark *Notorynchus cepedianus* . Marine Ecology Progress Series 442: 133–148.

[3] Simpfendorfer CA, Heupel MR, White WT, Dulvy NK (2011) The importance of research and public opinion to conservation management of sharks and rays: a synthesis. Marine and Freshwater Research 62: 518–527.

[4] Speed C, Meekan M, Field I, McMahon C, Abrantes K, et al (2012) Trophic ecology of reef sharks determined using stable isotopes and telemetry. Coral Reefs 31: 357–367.

[5] Myers RA, Baum JK, Shepherd TD, Powers SP, Peterson CH (2007) Cascading Effects of the Loss of Apex Predatory Sharks from a Coastal Ocean. Science 315: 1846–1850. 17395829 10.1126/science.1138657

[6] Ferretti F, Worm B, Britten GL, Heithaus MR, Lotze HK (2010) Patterns and ecosystem consequences of shark declines in the ocean. Ecology Letters 13: 1055–1071. 20528897 10.1111/j.1461-0248.2010.01489.x

[7] Heithaus MR, Wirsing AJ, Dill LM (2012) The ecological importance of intact top-predator populations: a synthesis of 15 years of research in a seagrass ecosystem. Marine and Freshwater Research 63: 1039–1050.

[8] Speed CW, Meekan MG, Field IC, McMahon CR, Stevens JD, et al (2011) Spatial and temporal movement patterns of a multi-species coastal reef shark aggregation. Marine Ecology Progress Series 429: 261–275.

[9] Worm B, Davis B, Kettemer L, Ward-Paige CA, Chapman D, et al (2013) Global catches, exploitation rates, and rebuilding options for sharks. Marine Policy 40: 194–204.

[10] Dulvy NK, Fowler SL, Musick JA, Cavanagh RD, Kyne PM, et al.. (2014) Extinction risk and conservation of the world's sharks and rays. eLife 3 : http://dx.doi.org/10.7554/eLife.00590 10.7554/eLife.00590 PMC3897121 24448405

[11] Compagno LJV (1984) Sharks of the world. An annotated and illustrated catalogue of shark species to date. Part II (*Carcharhiniformes*). FAO Fisheries Synopsis: 250–655.

[12] Cliff G, Dudley SFJ (1991) Sharks caught in the protective gill nets off Natal, South Africa. 4. The bull shark *Carcharhinus leucas* Valenciennes. South African Journal of Marine Science 10: 253–270.

[13] Daly R, Froneman PW, Smale MJ (2013) Comparative Feeding Ecology of Bull Sharks (*Carcharhinus leucas*) in the Coastal Waters of the Southwest Indian Ocean Inferred from Stable Isotope Analysis. PLoS ONE 8: e78229. 24205168 10.1371/journal.pone.0078229 PMC3804608

[14] Brunnschweiler JM, Queiroz N, Sims DW (2010) Oceans apart? Short-term movements and behaviour of adult bull sharks *Carcharhinus leucas* in Atlantic and Pacific Oceans determined from pop-off satellite archival tagging. Journal of Fish Biology 77: 1343–1358. 21039509 10.1111/j.1095-8649.2010.02757.x

[15] Carlson JK, Ribera MM, Conrath CL, Heupel MR, Burgess GH (2010) Habitat use and movement patterns of bull sharks *Carcharhinus leucas* determined using pop-up satellite archival tags. Journal of Fish Biology 77: 661–675. 20701646 10.1111/j.1095-8649.2010.02707.x

[16] Compagno LJV, Smale MJ (1986) Recent records of four warm-water elasmobranchs from the Eastern Cape province, South Africa. South African Journal of Marine Science 4: 11–15.

[17] Mann BQ (2000) Southern African Marine Linefish Status Reports. Oceanographic Research Institute Special Report: 265p.

[18] Wintner SP, Dudley SFJ, Kistnasamy N, Everett B (2002) Age and growth estimates for the Zambezi shark, *Carcharhinus leucas,* from the east coast of South Africa. Marine and Freshwater Research 53: 557–567.

[19] Smith SE, Au DW, Show C (1998) Intrinsic rebound potentials of 26 species of Pacific sharks. Marine and Freshwater Research 49: 663–678.

[20] Barnett A, Abrantes KG, Stevens JD, Semmens JM (2011) Site fidelity and sex-specific migration in a mobile apex predator: implications for conservation and ecosystem dynamics. Animal Behaviour 81: 1039–1048.

[21] Dudley SFJ, Simpfendorfer CA (2006) Population status of 14 shark species caught in the protective gillnets off KwaZulu-Natal beaches, South Africa, 1978–2003. Marine and Freshwater Research 57: 225–240.

[22] Warkentin IG, Hernández D (1996) The conservation implications of site fidelity: A case study involving nearctic-neotropical migrant songbirds wintering in a Costa Rican mangrove. Biological Conservation 77: 143–150.

[23] Shuter JL, Broderick AC, Agnew DJ, Jonzen N, Godley BJ, et al.. (2011) Conservation and management of migratory species. In: Milner-Gulland EJ, Fryxell JM, Sinclair ARE, editors. Animal migration: a synthesis: Oxford University Press. pp. 172–206.

[24] Turpie JK, Beckley LE, Katua SM (2000) Biogeography and the selection of priority areas for conservation of South African coastal fishes. Biological Conservation 92: 59–72.

[25] Sink K, Harris J, Lombard A (2004) South African National Spatial Biodiversity Assessment 2004: Technical Report Vol. 4 Marine Component 11.

[26] Ramsay PJ (1994) Marine geology of the Sodwana Bay shelf, southeast Africa. Marine Geology 120: 225–247.

[27] Riegl B, Schleyer MH, Cook PJ, Branch GM (1995) Structure of Africa's Southernmost Coral Communities. Bulletin of Marine Science 56: 676–691.

[28] Stevens JD (1999) Shark tagging: a brief history of methods. Fish Movement and Migration 65–68.

[29] Kessel ST, Cooke SJ, Heupel MR, Hussey NE, Simpfendorfer CA, et al.. (2013) A review of detection range testing in aquatic passive acoustic telemetry studies. Reviews in Fish Biology and Fisheries: 1–20.

[30] Welsh JQ, Fox RJ, Webber DM, Bellwood DR (2012) Performance of remote acoustic receivers within a coral reef habitat: implications for array design. Coral Reefs 31: 693–702.

[31] Brunnschweiler JM, Barnett A (2013) Opportunistic Visitors: Long-Term Behavioural Response of Bull Sharks to Food Provisioning in Fiji. PLoS ONE 8: e58522. 23516496 10.1371/journal.pone.0058522 PMC3596312

[32] How JR, de Lestang S (2012) Acoustic tracking: issues affecting design, analysis and interpretation of data from movement studies. Marine and Freshwater Research 63: 312–324.

[33] Batschelet E (1981) Circular statistics for biology. London Academic Press.

[34] Russell GS, Levitin DJ (1996) An expanded table of probability values for Rao's Spacing Test. Communications in Statistics: Simulation and Computation 24: 879–888.

[35] Bond ME, Babcock EA, Pikitch EK, Abercrombie DL, Lamb NF, et al (2012) Reef sharks exhibit site-fidelity and higher relative abundance in marine reserves on the Mesoamerican barrier reef. PLoS ONE 7: e32983. 22412965 10.1371/journal.pone.0032983 PMC3297616

[36] Welsh JQ, Bellwood DR (2012) How far do schools of roving herbivores rove? A case study using *Scarus rivulatus* . Coral Reefs 31: 991–1003.

[37] Dingle H, Drake VA (2007) What is migration? Bioscience 57: 113–121.

[38] Bansemer CS, Bennett MB (2011) Sex- and maturity-based differences in movement and migration patterns of grey nurse shark, *Carcharias taurus*, along the eastern coast of Australia. Marine and Freshwater Research 62: 596–606.

[39] Heupel MR, Yeiser BG, Collins AB, Ortega L, Simpfendorfer CA (2010) Long-term presence and movement patterns of juvenile bull sharks, *Carcharhinus leucas*, in an estuarine river system. Marine and Freshwater Research 61: 1–10.

[40] Barnett A, Abrantes KG, Seymour J, Fitzpatrick R (2012) Residency and Spatial Use by Reef Sharks of an Isolated Seamount and Its Implications for Conservation. PLoS ONE 7: e36574. 22615782 10.1371/journal.pone.0036574 PMC3353940

[41] Filmalter JD, Dagorn L, Cowley PD (2013) Spatial behaviour and site fidelity of the sicklefin lemon shark *Negaprion acutidens* in a remote Indian Ocean atoll. Marine Biology 160: 2425–2436.

[42] Heupel MR, Simpfendorfer C (2005) Quantitative analysis of aggregation behavior in juvenile blacktip sharks. Marine Biology 147: 1239–1249.

[43] DiGirolamo AL, Gruber SH, Pomory C, Bennett WA (2012) Diel temperature patterns of juvenile lemon sharks *Negaprion brevirostris*, in a shallow-water nursery. Journal of Fish Biology 80: 1436–1448. 22497392 10.1111/j.1095-8649.2012.03263.x

[44] Brunnschweiler JM, Baensch H (2011) Seasonal and Long-Term Changes in Relative Abundance of Bull Sharks from a Tourist Shark Feeding Site in Fiji. PLoS ONE 6: e16597. 21346792 10.1371/journal.pone.0016597 PMC3029404

[45] Weng KC, Castilho PC, Morrissette JM, Landeira-Fernandez AM, Holts DB, et al (2005) Satellite Tagging and Cardiac Physiology Reveal Niche Expansion in Salmon Sharks. Science 310: 104–106. 16210538 10.1126/science.1114616

[46] Papastamatiou YP, Meyer CG, Carvalho F, Dale J, Hutchinson M, et al (2013) Telemetry and random walk models reveal complex patterns of partial migration in a large marine predator. Ecology 94: 2595–2606. 24400511 10.1890/12-2014.1

[47] Werry JM, Planes S, Berumen ML, Lee KA, Braun CD, et al (2014) Reef-Fidelity and Migration of Tiger Sharks, *Galeocerdo cuvier*, across the Coral Sea. PLoS ONE 9: e83249. 24421879 10.1371/journal.pone.0083249 PMC3885424

[48] Fitzpatrick R, Thums M, Bell I, Meekan MG, Stevens JD, et al (2012) A comparison of the seasonal movements of tiger sharks and green turtles provides insight into their predator-prey relationship. PLoS ONE 7: e51927. 23284819 10.1371/journal.pone.0051927 PMC3526478

[49] Kock A, O'Riain MJ, Mauff K, Meÿer M, Kotze D, et al (2013) Residency, Habitat Use and Sexual Segregation of White Sharks, *Carcharodon carcharias*, in False Bay, South Africa. PLoS ONE 8: e55048. 23383052 10.1371/journal.pone.0055048 PMC3557240

[50] Holland KN, Wetherbee BM, Lowe CG, Meyer CG (1999) Movements of tiger sharks (*Galeocerdo cuvier*) in coastal Hawaiian waters. Marine Biology 134: 665–673.

[51] Bonfil R, Meyer M, Scholl MC, Johnson R, O'Brien S, et al (2005) Transoceanic migration, spatial dynamics and population linkages of White Sharks. Science 310: 100–103. 16210537 10.1126/science.1114898

[52] Heithaus MR, Wirsing AJ, Dill LM, Heithaus LI (2007) Long-term movements of tiger sharks satellite-tagged in Shark Bay, Western Australia. Marine Biology 151: 1455–1461.

[53] Hammerschlag N, Luo J, Irschick DJ, Ault JS (2012) A Comparison of Spatial and Movement Patterns between Sympatric Predators: Bull Sharks (*Carcharhinus leucas*) and Atlantic Tarpon (*Megalops atlanticus*). PLoS ONE 7: e45958. 23049904 10.1371/journal.pone.0045958 PMC3458817

[54] Holdo RM, Holt RD, Sinclair ARE, Godley BJ, Thirgood S (2011) Migration impacts on communities and ecosystems: empirical evidence and theoretical insights. In: Animal migration, a synthesis. Eds Milner-Gulland EJ, Fryxell JM and Sinclair ARE. Oxford University Press 2011. pp. 131–143.

