# Supplementary figures and images for: Residency Patterns and Migration Dynamics of Adult Bull Sharks (Carcharhinus leucas) on the East Coast of Southern Africa
Source: PLoS One. 2014 Oct 8;9(10):e109357. doi: 10.1371/journal.pone.0109357 (PMC4190266; doi:10.1371/journal.pone.0109357)

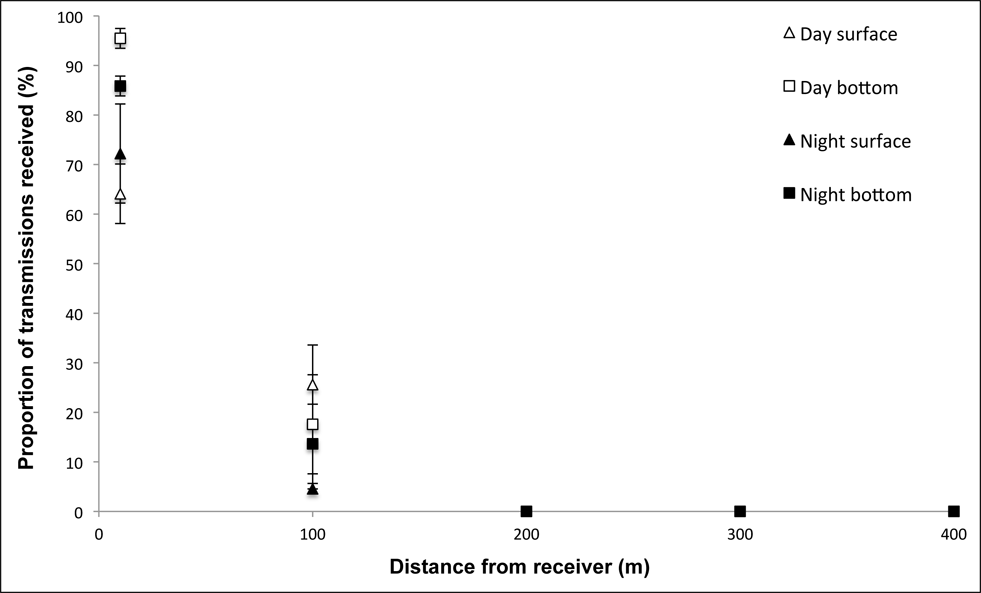

Supplement: Figure S1 — Range test results. Proportion of transmissions received vs the expected detections received represented as a percentage (0–100%) from tags deployed at the study site at varying distances, depths and times of day. Error bars represent standard deviation. (TIF) [file pone.0109357.s001.tif]
