# Supplementary material for: Residency Patterns and Migration Dynamics of Adult Bull Sharks (Carcharhinus leucas) on the East Coast of Southern Africa
Source: PLoS One. 2014 Oct 8;9(10):e109357. doi: 10.1371/journal.pone.0109357 (PMC4190266; doi:10.1371/journal.pone.0109357)
Supplement: Table S1 — Range test results represented as mean detection rates expressed as percentages. Distance corresponds to the distance of the receiver from the transmitter (5 m, 100 m, 200 m, 300 m, 400 m), Day or Night corresponds to the time of day detections were recorded and Surface (10 m) or Bottom (25 m) corresponds with the depth at which the transmitter was positioned in the water column. Standard deviations are presented in parenthesis. (DOCX) [file pone.0109357.s002.docx]

**Table S1. Range test results represented as mean detection rates expressed as percentages.** Distance corresponds to the distance of the receiver from the transmitter (5m, 100m, 200m, 300m, 400m), Day or Night corresponds to the time of day detections were recorded and Surface (10m) or Bottom (25m) corresponds with the depth at which the transmitter was positioned in the water column. Standard deviations are presented in parenthesis.

| Distance (m) | Day surface (%) | Day bottom (%) | Night surface (%) | Night bottom (%) |
| --- | --- | --- | --- | --- |
| 5m | 64.1 (5.6) | 95.5 (2.2) | 72.2 (10.1) | 85.9 (2.3) |
| 100 | 25.6 (8.1) | 17.6 (10.4) | 4.6 (7.8) | 13.6 (10.5) |
| 200 | 0 | 0 | 0 | 0 |
| 300 | 0 | 0 | 0 | 0 |
| 400 | 0 | 0 | 0 | 0 |
