# Supplementary material for: Residency Patterns and Migration Dynamics of Adult Bull Sharks (Carcharhinus leucas) on the East Coast of Southern Africa
Source: PLoS One. 2014 Oct 8;9(10):e109357. doi: 10.1371/journal.pone.0109357 (PMC4190266; doi:10.1371/journal.pone.0109357)
Supplement: Text S1 — Range test experiment. Methods, results and discussion of the range test experiment. (DOCX) [file pone.0109357.s003.docx]

**Range test experiment**

*Range test methods*

Range tests were conducted by positioning an acoustic transmitter (VEMCO V16-6L) at various distances from a receiver (5m, 100m, 200m, 300m, 400m) and depths (bottom = 25m deep, surface =10m deep) for a period of four days per interval and depth. When possible, environmental conditions (wind direction and strength, swell height, sea surface temperature, current direction and strength) at the study site were recorded during the range test. Detections recorded by each receiver were grouped into bins representing “day” and “night” hours. Individual detection probabilities were then calculated for each period (day and night) at each position (surface and bottom) from each distance (5m, 100m, 200m, 300m, 400m) based on the total number of recorded detections expressed as a percentage (0-100%) of the known number of transmissions from the tag. The mean detection rates at each time of day, depth and distance were compared using a one-way ANOVA.

*Range test results*

Summary results from the range test experiment are presented in Table S1 and Figure S1. Combined mean detection rates at 5m were 79.5% (SD=14.0), at 100m were 15.4% (SD=8.7) and at 200m no detections were recorded. Detection rates were significantly higher (ANOVA, DF=5, p<0.05) during the day than at night when the tag transmitted from the bottom position (25m deep) at 5m and 100m distance from the receivers. The detection rate was also significantly higher (ANOVA, DF=5, p<0.05) during the day compared to night when the tag transmitted from the surface (10m deep) at 100m from the receiver but there was no significant difference (ANOVA, DF=5, p<0.05) between day and night when the tag transmitted from a distance of 5m. Bottom (25m deep) tag detection rates were significantly higher (ANOVA, DF=5, p<0.05) than surface detection rates (10m deep) during the day and night when the tag transmitted from a distance of 5m but there was no significant difference (ANOVA, DF=5, p<0.05) in detection rates when the tag transmitted from 100m distance during day or night. In all cases there was a significantly higher (ANOVA, DF=5, p<0.05) detection rate for all tags transmitting from 5m compared to 100m.

Environmental conditions recorded during the range test varied widely with wind direction (north, northeast, east, south and southwest), wind strength (5-25 knots), swell height (1.5-3m), current direction (north, south) and current strength (0 to >2 knots) all typical for the study site.

*Range test discussion*

Receiver range testing was conducted at the Pinnacle reef to help interpret the detection data at the study site [1,2]. Results from the range test experiment suggested that the detection range of receivers in this study was relatively poor [2,3] although within the same range as previously reported. Previous studies [2,4] using the same tag specifications (VEMCO V16-6L) in similar depths to our study reported mean detections rates of less than 60m [4] and 80% detection rates at 0m which were reduced to approximately 35% at 100m and 10% at 200m [2]. In comparison, we found that at 5m from the receiver we had combined mean detection rates of 79.5% and at 100m the mean detection rate was reduced to 15.4% and at 200m we received no detections. Although due to the scale of our experiment it is not possible to determine the actual detection threshold and caution needs to be taken when interpreting our results. Our range test experiment did suggest that increased biological noise from the Pinnacle reef at night may have negatively impacted the detection range of transmitters, especially when the tag transmitted from a position near to the reef. This was expected as nocturnal biological reef noise has been known to lower detection rates, specifically in tropical environments due to increased acoustic interference [3]. Reef rugosity and profile at the study site may have further compounded detection interference when tags transmitted close to the reef as acoustic signals may have been reflected or refracted by the reef [3]. Surface wave and wind action may also negatively impact detection rates due to increased acoustic interference but the range test experiment did not adequately control for the potential interference of absolute transmitter and receiver distance from surface interference.

Although the range test experiment showed that the detection range at the study site was poor, the actual detection range and performance of the acoustic receiver array at the study site appeared to be adequate for the study subject. This is apparent when investigating the actual detection data from tagged sharks that showed some sharks (44%) were detected by the receiver array at the Pinnacle reef for continuous periods of between 46 and 158 days. Periods of shark absence are further confirmed by detections from locations other than the study site for 10 out of 18 tagged sharks. Based on 111 observations at our study site of bull shark behavior it was evident that sharks swam slowly relatively close the reef and had a preference for shallow reef areas. This suggests that the most important detection interference may come from reef noise and profile but the sharks slow swimming speed and preference for shallow areas where receivers were located are important considerations that may have improved detection rates. In our study the range test experiment gave us a proxy for receiver performance but the actual receiver performance is still largely dependent on the study subject and associated behavior relative to the site-specific acoustic receiver array.

**References**

1. Kessel ST, Cooke SJ, Heupel MR, Hussey NE, Simpfendorfer CA, et al. (2013) A review of detection range testing in aquatic passive acoustic telemetry studies. Reviews in Fish Biology and Fisheries: 1-20.

2. How JR, de Lestang (2012) Acoustic tracking: issues affecting design, analysis and interpretation of data from movement studies. Marine and Freshwater Research 63: 312-324.

3. Welsh JQ, Fox RJ, Webber DM, Bellwood DR (2012) Performance of remote acoustic receivers within a coral reef habitat: implications for array design. Coral Reefs 31: 693-702.

4. Brunnschweiler JM, Barnett A (2013) Opportunistic Visitors: Long-Term Behavioural Response of Bull Sharks to Food Provisioning in Fiji. PLoS ONE 8: e58522.
